# Xpert MTB/RIF Ultra for the Diagnosis of Tuberculous Meningitis: A Small Step Forward

**DOI:** 10.1093/cid/ciaa473

**Published:** 2020-06-16

**Authors:** Joseph Donovan, Fiona V Cresswell, Nguyen Thuy Thuong Thuong, David R Boulware, Guy E Thwaites, Nathan C Bahr, Tuberculous Meningitis International Research Consortium, Rob E Aarnoutse, Suzanne T B Anderson, Nathan C Bahr, Nguyen D Bang, David R Boulware, Tom Boyles, Lindsey H M te Brake, Satish Chandra, Felicia C Chow, Fiona V Cresswell, Reinout van Crevel, Angharad G Davis, Sofiati Dian, Joseph Donovan, Kelly E Dooley, Anthony Figaji, A Rizal Ganiem, Ravindra Kumar Garg, Diana M Gibb, Raph L Hamers, Nguyen T T Hiep, Darma Imran, Akhmad Imron, Sanjay K Jain, Sunil K Jain, Jayantee Kalita, Rashmi Kumar, Vinod Kumar, Arjan van Laarhoven, Rachel P-J Lai, Abi Manesh, Suzaan Marais, Vidya Mave, Graeme Meintjes, David B Meya, Usha K Misra, Manish Modi, Alvaro A Ordonez, Nguyen H Phu, Sunil Pradhan, Kameshwar Prasad, Alize M Proust, Lalita Ramakrishnan, Ursula Rohlwink, Rovina Ruslami, Johannes F Schoeman, James A Seddon, Kusum Sharma, Omar Siddiqi, Regan S Solomons, Nguyen T T Thuong, Guy E Thwaites, Ronald van Toorn, Elizabeth W Tucker, Sean A Wasserman, Robert J Wilkinson

**Affiliations:** 1 Oxford University Clinical Research Unit, Centre for Tropical Medicine, Ho Chi Minh City, Viet Nam; 2 Centre for Tropical Medicine and Global Health, Nuffield Department of Medicine, University of Oxford, Oxford, United Kingdom; 3 Clinical Research Department, London School of Hygiene and Tropical Medicine, London, United Kingdom; 4 Infectious Diseases Institute, College of Health Sciences, Makerere University, Kampala, Uganda; 5 Medical Research Council/Uganda Virus Research Institute and London School of Hygiene and Tropical Medicine Uganda Research Unit, Entebbe, Uganda; 6 Division of Infectious Diseases and International Medicine, Department of Medicine, University of Minnesota, Minneapolis, Minnesota, USA; 7 Division of Infectious Diseases, Department of Medicine, University of Kansas, Kansas City, Kansas, USA

**Keywords:** tuberculous meningitis, diagnosis, Ultra, Xpert, cerebrospinal fluid

## Abstract

The delayed diagnosis of tuberculous meningitis (TBM) leads to poor outcomes, yet the current diagnostic methods for identifying *Mycobacterium tuberculosis* in cerebrospinal fluid (CSF) are inadequate. The first comparative study of the new GeneXpert MTB/RIF Ultra (Xpert Ultra) for TBM diagnosis suggested increased sensitivity of Xpert Ultra. Two subsequent studies have shown Xpert Ultra has improved sensitivity, but has insufficient negative predictive value to exclude TBM. Collecting and processing large volumes of CSF for mycobacterial testing are important for optimal diagnostic test performance. But clinical, radiological, and laboratory parameters remain essential for TBM diagnosis and empiric therapy is often needed. We therefore caution against the use of Xpert Ultra as a single diagnostic test for TBM; it cannot be used to “rule out” TBM.

## Teaser

Xpert Ultra may be superior to Xpert for detecting *Mycobacterium tuberculosis* in cerebrospinal fluid. However, Xpert Ultra has insufficient negative predictive value to exclude Tuberculous Meningitis (TBM). We caution against using Xpert Ultra as a single diagnostic test for TBM.

Tuberculous meningitis (TBM) is the most severe form of tuberculosis (TB), leading to death in 30–50% of individuals despite treatment [1–4]. Early diagnosis of TBM is vital and delayed diagnosis leads to poor clinical outcomes [5]. Confirming a TBM diagnosis requires identification of *Mycobacterium tuberculosis* (Mtb) in cerebrospinal fluid (CSF). However, unlike sputum in pulmonary TB, there are few bacteria in the CSF, and Mtb detection is often challenging. Conventional TBM diagnosis depends upon CSF Ziehl-Neelsen (ZN) smear microscopy to detect acid-fast bacilli (AFB), CSF mycobacterial culture, and, if available, detection of mycobacterial DNA in CSF. Each test modality has advantages and limitations. Ziehl-Neelsen smear microscopy can be performed quickly and requires minimal specialist equipment, yet it is often insensitive and true positive cases are frequently missed [6]. ZN smear sensitivity is influenced by CSF processing steps and microscopist expertise, with sensitivities of usually 10–50% [7–9] and rarely higher [10, 11]. Mycobacterial culture, when positive, allows drug susceptibility testing; however, results are usually not available for at least 2 weeks, too late to guide early anti-TB chemotherapy. In contrast, fully automated polymerase chain reaction (PCR) testing with Xpert MTB/RIF (Xpert) testing using the GeneXpert platform (Cepheid, Sunnyvale, CA) can generate results in under 2 hours and, if positive, includes rifampicin resistance prediction. While generally cheaper than culture techniques, the Xpert per cartridge cost is approximately $10 in countries eligible for concessional pricing, and cartridge provision remains heavily supported by donor organizations in many settings. In recent TBM studies [9, 11–14], sensitivity of mycobacterial culture ranged from 26% to 67%, and sensitivity of Xpert ranged from 18% to 59%. Xpert has been rapidly adopted worldwide largely due to its speed and ease of use allowing for reduced reliance upon time-consuming smear microscopy by experienced technicians. While some training is required to run the Xpert instrument, much less expertise and time are required than are needed to accurately diagnose TBM by smear microscopy—few in the world have mastered this skill. Neither of these tests is adequate for TBM diagnosis, and so a high degree of clinical suspicion and a low threshold to initiate empiric anti-TB chemotherapy are crucial to successful outcomes from TBM treatment. All currently available diagnostic tests for TBM rely on CSF sampling, which presents a barrier to improving TBM diagnosis, as CSF sampling may be delayed, unavailable or contraindicated in some settings.

To address limitations with Xpert for TB diagnosis, the Xpert MTB/RIF Ultra (Xpert Ultra) cartridge was developed [15]. Modifications in the Xpert Ultra cartridge include a larger reaction chamber to double the amount of sample, and thereby DNA, tested, as well as incorporation of 2 additional multicopy amplification target genes (IS6110 and IS1081) [15]. In contrast to the single *rpoB* gene, the IS6110 and IS1081 genes appear in variable numbers between Mtb lineages and within a single lineage [15, 16]. These cartridge modifications aimed to improve diagnostic sensitivity and improve reliability of rifampicin-resistance detection. In vitro, the lower limit of detection decreased to approximately 16 colony forming units (CFU)/mL from approximately 100–120 CFU/mL for Xpert, similar to culture (~10 CFU/mL) [15].

Complicating efforts to improve TBM diagnosis is the fact that no single reference “gold standard” test exists. Typically, new tests are measured against mycobacterial culture alone, composite reference standards (positive ZN smear microscopy, Xpert or mycobacterial culture), or consensus clinical case definitions [17]. A composite reference standard may represent all microbiologically confirmed TBM, not just that confirmed by mycobacterial culture, as culture is known to be only moderately sensitive. Inclusion of Xpert Ultra (the index test) in a composite reference standard aims to more accurately estimate Xpert Ultra sensitivity (ie, it allows samples positive only by Xpert Ultra to be in the reference standard, with an assumption that the likelihood of false-positive Xpert Ultra tests is very low). However, Xpert Ultra specificity cannot be assessed with this approach (specificity would always be 100%; all tests would be true positives). Each reference standard has its own issues and presenting a combination of these possibilities gives the reader the most complete picture of test performance, albeit with caveats. Regardless of the standard(s) used, it is clear that cases of TBM are being missed with currently available diagnostic tests. It is against this range of reference standards that Xpert Ultra has been evaluated.

The World Health Organization (WHO) recommended that Xpert Ultra replace Xpert in all settings in March 2017 [18]. This followed on from a 2014 WHO Xpert implementation manual [19], which strongly recommended that “Xpert should be used in preference to conventional microscopy and culture as the initial diagnostic test for cerebrospinal fluid (CSF).” After the 2014 WHO recommendation regarding Xpert for TBM, the Tuberculous Meningitis International Research Consortium authored a statement conveying that, while it was reasonable to use Xpert as the first test for TBM, it should not be the last test, as Xpert was not sufficiently accurate to “rule out” TBM [20].

The initial study of Xpert Ultra for TBM diagnosis was promising. Among 129 Ugandan adults with human immunodeficiency virus (HIV) with suspected meningitis (23 with definite or probable TBM), diagnostic sensitivities of Xpert Ultra, Xpert, and mycobacterial culture were 69.6% (95% confidence interval [CI], 47.1–86.8%), 43.5% (95% CI, 23.2–65.5%), and 43.5% (95% CI, 23.2–65.5%) [14]. Against a composite microbiological reference standard (Xpert, culture, or Xpert Ultra), Xpert Ultra sensitivity was 95.5% versus 45.5% for culture or Xpert. Importantly, even in the best-case scenario (the composite microbiological reference standard), Xpert Ultra did not detect all TBM cases.

In January 2020, 2 larger prospective studies evaluating Xpert Ultra were published. In the study by Cresswell et al [13], 204 Ugandan adults (96% coinfected with HIV) with suspected meningitis had CSF Xpert Ultra performed. Compared with a reference of definite or probable TBM, test sensitivities were 76.5% (95% CI, 62.5–87.2%) for Xpert Ultra, 55.6% (95% CI, 44.0–70.4%) for Xpert, and 61.4% (95% CI, 45.5–75.6%) for mycobacterial culture. In this study “possible TBM” cases were not included in the reference standard as this category is nonspecific in HIV coinfection due to concomitant brain pathologies associated with advanced immunosuppression. In the second study, Donovan et al [12] randomized 205 Vietnamese adults (15% coinfected with HIV) with meningitis to either Xpert Ultra or Xpert testing. Against a reference standard of definite, probable, or possible TBM, test sensitivities were 47.2% (95% CI, 34.4–60.3%) for Xpert Ultra, 39.6% (95% CI, 27.6–53.1%) for Xpert, and 47.9% (95% CI, 38.0–57.9%) for mycobacterial culture. As with Xpert [11], specificity of Xpert Ultra for TBM diagnosis was high in both studies [12, 13]. Xpert Ultra sensitivity was statistically superior to that of Xpert in Uganda but not in a predominantly HIV-negative Vietnam population. The TBM diagnostic studies using Xpert Ultra are summarized in Table 1.

**Table 1.** Summary of Tuberculous Meningitis Diagnostic Studies Using Xpert MTB/RIF Ultra

First Author, Year of Publication	Location	Type of Study	HIV Infection, % (n/N)	Reference Standard(s) (No. of TBM Cases)	Xpert Ultra Sensitivity, % (n/N)	Negative Predictive Value, % (n/N)
Donovan et al [12], 2020	Vietnam	Randomised, prospective diagnostic study of meningitis suspects	25 (27/108)	Definite, probable, possible TBM (n = 108)	47 (25/53)	61 (44/72)
				Definite, probable TBM (n = 88)	58 (25/43)	75 (54/72)
				Definite TBM (n = 82)	60 (25/42)	76 (55/72)
				Positive mycobacterial culture (n = 45)	91 (20/22)	97 (62/64)
Cresswell et al [13], 2020	Uganda	Prospective cohort of meningitis suspects	98 (50/51)	Definite, probable TBM (n = 51)	77 (39/51)	93 (153/165)
			98 (41/42)	Composite microbiologic standard (n = 42)	93 (39/42)	98 (153/156)
Wang et al [21], 2019	China	Prospective cohort in paucibacillary TB (inclusive of TBM)^a^	0 (0/43)	Definite, probable, possible TBM (n = 43)	44 (19/43)	42 (17/41)
				Composite microbiologic standard (n = 22)	86 (19/22)	NA
Bahr et al [14], 2018	Uganda	Prospective cohort with retrospective CSF testing of meningitis suspects	100 (23/23)	Definite, probable TBM (n = 23)	70 (16/23)	94 (100/107)
				Composite microbiologic standard (n = 22)	96 (21/22)	99 (106/107)
Wu et al [22], 2019	China	Prospective diagnostic study in extrapulmonary TB (inclusive of TBM)^a^	0 (0/16)	Composite microbiologic standard (n = 16)	13 (2/16)	NA
Chin et al [23], 2019	Uganda	Case series of testing in suspected TBM	18 (2/11)	Suspected TBM (n = 11)	64 (7/11)	NA
Perez-Risco et al [24], 2018	Spain	Evaluation of smear-negative extrapulmonary samples (inclusive of TBM)^a^	Not stated	Positive mycobacterial culture (n = 3)	100 (3/3)	NA

Abbreviations: CSF, cerebrospinal fluid; HIV, human immunodeficiency virus; NA, not available; TB, tuberculosis; TBM, tuberculous meningitis.

^a^For studies of paucibacillary or extrapulmonary TB, data shown only for TBM cases. Definite, probable, or possible TBM defined per the research uniform case definition [17]. Composite microbiologic standard = positive by CSF testing of microscopy, Xpert MTB/RIF, Xpert MTB/RIF Ultra, or mycobacterial culture.

What can be learned from these studies? First, diagnostic tests cannot be expected to perform identically in all settings. Differences in tested CSF volume, CSF processing, HIV coinfection, genetics influencing host response to Mtb, and Mtb lineages could all contribute to these different results, as could the differences in study design (eg, dividing specimens among the tests vs randomizing samples) and smear microscopy sensitivity. Second, regardless of the differences in the exact performance of Xpert Ultra, the most important point is that, while Xpert Ultra seems to be some improvement on Xpert, its negative predictive value is not sufficiently high to exclude TBM when the result is negative. Following on from the opinion piece published by Bahr et al [20] in 2016, we wish to caution against the use of Xpert Ultra or Xpert as single diagnostic tests for TBM. Focus should be placed on collecting and processing large volumes of CSF (>6 mL, just for mycobacterial testing), maximizing the number of Mtb bacteria in the tested sample, and improving chances of confirming a diagnosis of TBM. Centrifugation of CSF (3000 *g* for 15 minutes) concentrates Mtb in the pellet and improves diagnostic sensitivity [8]. To optimize the performance of ZN smear microscopy, an appropriate time should be spent reading CSF smear slides before considering them negative. Microscopist skill and experience are hugely important, although they are hard to quantify.

Clinical, radiological, and laboratory parameters remain essential. Symptoms of meningitis for more than 5 days [17] should lead to suspicion of TBM, and focal neurological deficits are common. Chest radiograph may demonstrate miliary or pulmonary TB, and brain imaging may show evidence of cerebral infarcts, basal meningeal enhancement, or hydrocephalus. Where CSF testing fails, diagnostic testing of other potentially involved sites may provide microbiological evidence of TB elsewhere. For example, sputum Xpert may detect Mtb, or urine TB-lipoarabinomannan (TB-LAM) may provide evidence of disseminated TB in patients coinfected with HIV. Lymphocytic, low-glucose, high-protein CSF is classically seen in TBM but is not always present and exclusion of cryptococcal meningitis is essential in immunosuppressed patients [25]. Repeat lumbar puncture after 48–72 hours may be valuable in patients who fail to improve with routine antibiotics, or in whom diagnosis remains unclear.

Tuberculous meningitis remains a devastating disease, and advances in the diagnostic field are being made. While Xpert Ultra represents a step forward in TBM diagnosis, Xpert Ultra cannot fully exclude this disease. Host biomarkers and antigen detection from CSF may have a future role in TBM diagnosis, but more studies are needed [26, 27]. Studies of Xpert Ultra for the diagnosis of pediatric TBM are also needed. The Tuberculous Meningitis International Research Consortium, which includes clinicians, basic scientists, and clinical pharmacologists, continues to meet regularly, most recently in March 2019 in Lucknow, India, to advance the TBM research and policy agenda [28]. Policy makers, while adopting improved technologies, must resist the temptation to point to any single test as a perfect tool. It is crucial that while adopting Xpert Ultra, clinicians keep in mind that this test is not perfect and cannot “rule out” TBM. The search for new and improved diagnostic tests must go on.

## Notes

***Tuberculous Meningitis International Research Consortium.*** Rob E. Aarnoutse, Suzanne T. B. Anderson, Nathan C. Bahr, Nguyen D. Bang, David R. Boulware, Tom Boyles, Lindsey H. M. te Brake, Satish Chandra, Felicia C. Chow, Fiona V. Cresswell, Reinout van Crevel, Angharad G. Davis, Sofiati Dian, Joseph Donovan, Kelly E. Dooley, Anthony Figaji, A. Rizal Ganiem, Ravindra Kumar Garg, Diana M. Gibb, Raph L. Hamers, Nguyen T. T. Hiep, Darma Imran, Akhmad Imron, Sanjay K. Jain, Sunil K. Jain, Jayantee Kalita, Rashmi Kumar, Vinod Kumar, Arjan van Laarhoven, Rachel P.-J. Lai, Abi Manesh, Suzaan Marais, Vidya Mave, Graeme Meintjes, David B. Meya, Usha K. Misra, Manish Modi, Alvaro A. Ordonez, Nguyen H. Phu, Sunil Pradhan, Kameshwar Prasad, Alize M. Proust, Lalita Ramakrishnan, Ursula Rohlwink, Rovina Ruslami, Johannes F. Schoeman, James A. Seddon, Kusum Sharma, Omar Siddiqi, Regan S. Solomons, Nguyen T. T. Thuong, Guy E. Thwaites, Ronald van Toorn, Elizabeth W. Tucker, Sean A. Wasserman, Robert J. Wilkinson.

***Acknowledgments*.** The authors acknowledge the organizers of the Tuberculous Meningitis International Research Consortium meeting and the Wellcome Trust for financially supporting the 2019 meeting.

***Financial support.*** This work was supported by the Wellcome Trust (grant number 106680/Z/14/Z; to G.E.T). The grant supports this work and the work of J. D., N. T. T. T., and G. E. T. F. V. C. is supported by a Wellcome Clinical PhD Fellowship in Global Health (grant number 210772/Z/18/2). D. R. B. and N. C. B. report funding from the National Institutes of Health (grant numbers K23 NS110470, R01 NS086312).

***Potential conflicts of interest.*** The authors: No reported conflicts of interest. All authors have submitted the ICMJE Form for Disclosure of Potential Conflicts of Interest.
